# The genetic diversity of *Oncomelania hupensis robertsoni*, intermediate hosts of *Schistosoma japonicum* in hilly regions of China, using microsatellite markers

**DOI:** 10.1186/s13071-024-06227-3

**Published:** 2024-03-21

**Authors:** Jing Song, Hongqiong Wang, Shizhu Li, Chunhong Du, Peijun Qian, Wenya Wang, Meifen Shen, Zongya Zhang, Jihua Zhou, Yun Zhang, Chunying Li, Yuwan Hao, Yi Dong

**Affiliations:** 1 Department of Schistosomiasis Control and Prevention, Yunnan Institute of Endemic Disease Control and Prevention, Dali, 671000 China; 2 Yunnan Key Laboratory of Natural Focus Disease Control Technology, Dali, 671000 China; 3 National Key Laboratory of Intelligent Tracking and Forecasting for Infectious Diseases, Chinese Center for Tropical Diseases Research; NHC Key Laboratory of Parasite and Vector Biology; WHO Collaborating Center for Tropical Diseases; National Center for International Research on Tropical Diseases, National Institute of Parasitic Diseases at Chinese Center for Disease Control and Prevention, Shanghai, 200025 China; 4 School of Global Health, Chinese Center for Tropical Diseases Research-Shanghai Jiao Tong University School of Medicine, Shanghai, China; 5 School of Public Health, Kunming Medical University, Kunming, 650500 China

**Keywords:** *Oncomelania hupensis*, *Oncomelania hupensis robertsoni*, Microsatellite DNA, Genetic diversity, *Schistosome japonicum*

## Abstract

**Background:**

The elimination of schistosomiasis remains a challenging task, with current measures primarily focused on the monitoring and control of *Oncomelania hupensis* (O. hupensis) snail, the sole intermediate host of Schistosome japonicum. Given the emerging, re-emerging, and persistent habitats of snails, understanding their genetic diversity might be essential for their successful monitoring and control. The aims of this study were to analyze the genetic diversity of *Oncomelania hupensis robertsoni* (O. h. robertsoni) using microsatellite DNA markers; and validate the applicability of previously identified microsatellite loci for O. hupensis in hilly regions.

**Methods:**

A total of 17 populations of *O. h. robertsoni* from Yunnan Province in China were selected for analysis of genetic diversity using six microsatellite DNA polymorphic loci (P82, P84, T4-22, T5-11, T5-13, and T6-27).

**Results:**

The number of alleles among populations ranged from 0 to 19, with an average of 5. The average ranges of expected (He) and observed (Ho) heterozygosity within populations were 0.506 to 0.761 and 0.443 to 0.792, respectively. The average fixation index within the population ranged from – 0.801 to 0.211. The average polymorphic information content (PIC) within the population ranged from 0.411 to 0.757, appearing to be polymorphic for all loci (all PIC > 0.5), except for P28 and P48. A total of 68 loci showed significant deviations from Hardy-Weinberg equilibrium (*P* < 0.05), and pairwise Fst values ranged from 0.051 to 0.379. The analysis of molecular variance indicated that 88% of the variation occurred within snail populations, whereas 12% occurred among snail populations. Phylogenetic trees and principal coordinate analysis revealed two distinct clusters within the snail population, corresponding to “Yunnan North” and “Yunnan South”.

**Conclusions:**

*O. h. robertson*i exhibited a relatively high level of genetic differentiation, with variation chiefly existing within snail populations. All snail in this region could be separated into two clusters. The microsatellite loci P82 and P84 might not be suitable for classification studies of O. hupensis in hilly regions. These findings provided important information for the monitoring and control of snail, and for further genetic diversity studies on snail populations.

**Graphical Abstract:**

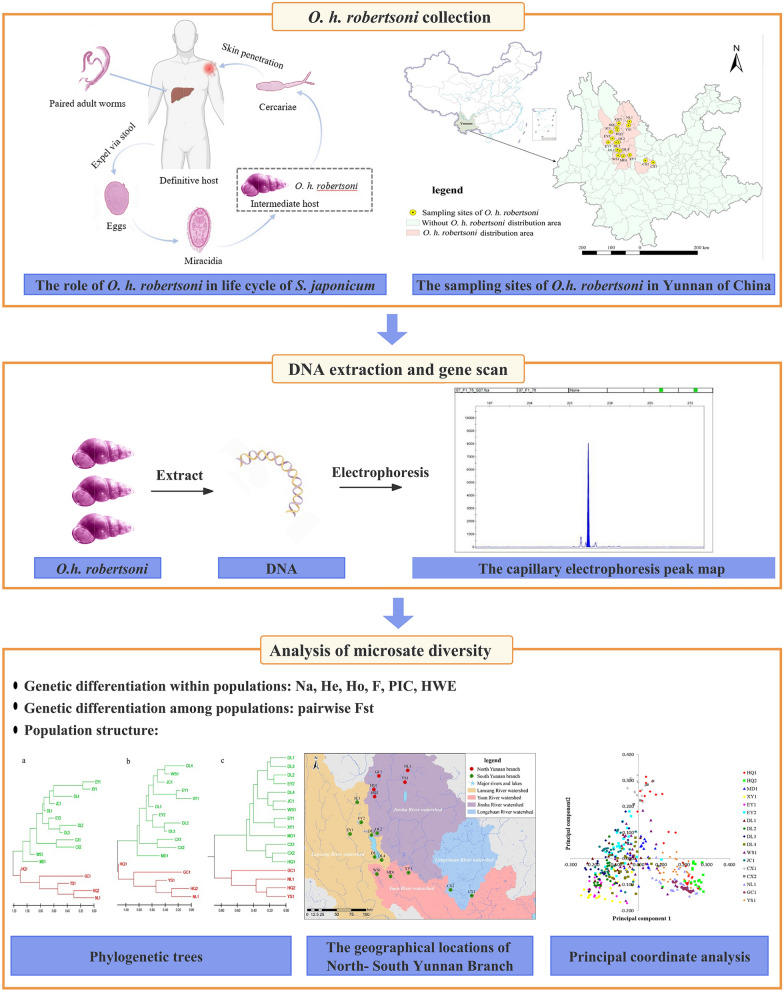

**Supplementary Information:**

The online version contains supplementary material available at 10.1186/s13071-024-06227-3.

## ﻿Background

Schistosomiasis is a neglected tropical parasitic disease that has imposed a severe burden worldwide, affecting almost 240 million people [1]. It is a disease endemic to China, where the causative agent is *Schistosoma japonicum*. After decades of major efforts in schistosomiasis control, substantial progress has been achieved. For example, the estimated number of infected humans was reduced from approximately 11.6 million in the 1950s to 37,601 in 2017 [2]. Thus, aligning with the updated targets set by the World Health Organization, the Chinese central government proposed the goal of eliminating schistosomiasis transmission by 2030 [3]. Despite this progress and ambitious goals, several major challenges remain, one of which is the emerging, re-emerging, and persistent habitats of *Oncomelania hupensis*, the only intermediate host snails of *S. japonicum* [4–6].

In mainland China, *O. hupensis* is primarily distributed in the drainage basins of the middle and lower reaches of the Yangtze River across 12 provinces, encompassing four subspecies: *Oncomelania hupensis hupensis*, *O. h. robertsoni*, *O. h. tangi* and *O. h. guangxiensis* [4]. *Oncomelania hupensis* populations in different regions exhibit diverse morphological and genetic characteristics, evolving in distinct directions [7]. Previous studies have highlighted regional variations in snail morphology, genetic characteristics, and susceptibility to *S. japonicum* [8]. Yunnan Province was previously a severe hotspot for schistosomiasis endemism in hilly regions of China [9] and still harbors a substantial population of snails, i.e. *O. h. robertsoni* [10]. Population genetic experiments have revealed that Yunnan Province was the likely place of origin for *O. hupensis* in mainland China [11, 12]. Smooth-shelled *O. hupensis*, introduced from India through Yunnan, gradually migrated eastward to the Yangtze River Watershed. During this process, genetic mutations occurred, giving rise to ribbed-shelled *O. hupensis*. Therefore, studying the genetic diversity of *O. h. robertsoni* is crucial for tracing the origin of *O. hupensis* in the Chinese mainland. Currently, *O. h. robertsoni* is predominantly found in Yunnan Province in areas with elevations ranging from 1350 to 2466 m, a region that represents the highest altitude endemic area in China. Additionally, the breeding environment of this population is highly complex, occurring in ditches, grassland, field ridges, wasteland, and dry land. Its distribution is relatively isolated and patchy, with substantial fragmentation [13, 14]. High mountain barriers or other forms of isolation exist between some distribution areas, creating geographically separated and non-contiguous regions [15]. Given this unique geographical environment, the genetic diversity of *O. h. robertsoni* could exhibit a certain degree of heterogeneity, posing challenges to monitoring and controlling these snails [16–18]. Therefore, fully understanding the genetic diversity of *O. h. robertsoni* is crucial, especially concerning snail control through focal molluscicides [19].

Microsatellite DNA is a short nucleotide tandem (STR) or simple sequence repeat (SSR) DNA, which is widely distributed in the genomes of eukaryotic organisms [20]. Given its characteristics of easy detection in populations, high heterozygosity, codominance, and wide distribution, microsatellite DNA has become a crucial molecular marker for studying population genetic diversity [21, 22]. Although the microsatellite DNA library of *O. hupensis* has been established [20, 23], few studies have used microsatellite markers to analyze the population genetic diversity and genome mapping of this species. Moreover, previous studies have primarily focused on *O. hupensis* from marshland and/or lake areas endemic for schistosomiasis in China [6, 20, 24], and there is a lack of population genetic analyses using microsatellites for *O. hupensis* population in schistosomiasis-endemic areas in hilly regions.

Previous studies have revealed the existence of *O. h. robertsoni* based on morphological characteristics, biological traits, and molecular markers [4]. This subspecies inhabits hilly schistosomiasis-endemic regions in both Yunnan and Sichuan Provinces and displays significant genetic variation compared with *O. hupensis* in the Yangtze River Watershed [25, 26]. The suitability of using the same microsatellite DNA loci for *O. hupensis* in hilly regions remains unknown, and this merits more research attention.

Therefore, this study aimed to analyze the genetic diversity of *O. h. robertsoni* using microsatellite DNA markers and to validate the applicability of previously identified microsatellite loci for *O. hupensis* in hilly regions. Our results may provide important information for monitoring and control of snails and further genetic diversity studies on snail populations.

## Methods

### Sources of *O. hupensis*

**Fig. 1:**
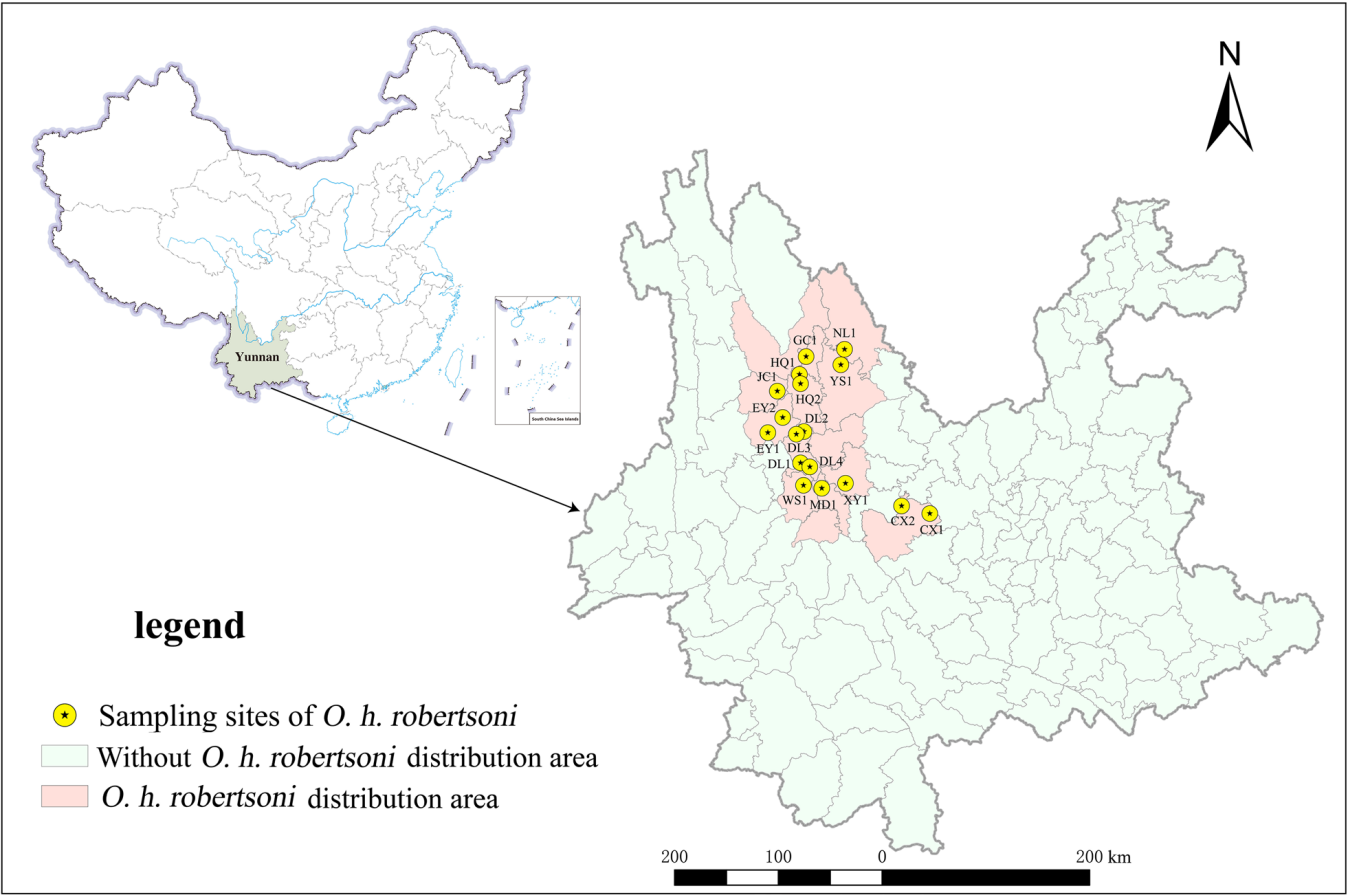
Distribution of sampling sites of *Oncomelania hupensis robertsoni* in Yunnan Province of China

A total of 17 populations of *O. h. robertsoni* were collected from formerly endemic schistosomiasis villages in Yunnan Province of China considering various factors such as snail density, geographical location, altitude, water system, and environmental type, etc. (Table 1, Fig. 1).

**Table 1:** Location of *Oncomelania hupensis robertsoni* collection

Collection site (code)	Habitat environment	No. samples	Collection date	Latitude	Longitude	Altitude (m)
Lianyi village (HQ1)	Ditch	110	3 Sept 2022	100.1825° E	26.6514° N	2213.00
Jinsuo village (HQ2)	Ditch	121	3 Sept 2022	100.1947° E	26.5436° N	2190.00
Caizhuang village (MD1)	Ditch	111	15 Sept 2022	100.4384° E	25.3431° N	1666.48
Xiaoqiao village (XY1)	Ditch	100	24 Sept 2022	100.7120°E	25.3989° N	1915.78
Qiandian village (EY1)	Vegetable field	113	29 Sept 2022	99.8189° E	25.9810° N	1907.34
Yongle village (EY2)	Ditch	106	29 Sept 2022	99.9901° E	26.1565° N	2042.25
Liuguanchang (DL1)	Ditch	100	7 Jan 2022	100.1944° E	25.6338° N	1979.20
Wuxing village (DL2)	Ditch	130	7 Jan 2022	100.2341° E	25.9937° N	1745.19
Xiaocen village (DL3)	Ditch	101	7 Jan 2022	100.1465° E	25.9645° N	1930.84
Fengming (DL4)	Ditch	100	7 Jan 2022	100.3021° E	23.5880° N	1998.20
Dianzhong village (WS1)	Ditch	104	12 Jan 2022	100.2290° E	25.3773° N	1717.82
Huilong (JC1)	Vegetable field	110	16 Jan 2022	99.9283° E	26.4579° N	2204.00
Cangling (CX1)	Grassland	100	20 Jan 2022	101.6801° E	25.0512° N	1807.30
Luhe (CX2)	Grassland	100	20 Jan 2022	101.3553° E	25.1392° N	1830.41
Biyuan (NL1)	Ditch	95	22 Jan 2022	100.7008° E	26.9380° N	2123.40
Dongyuan (GC1)	Grassland	97	25 Jan 2022	100.2596° E	26.8549° N	2336.20
Yangwu (YS1)	Ditch	102	29 Jan 2022	100.6582° E	26.7611° N	1562.00

*HQ* Heqing County; *MD* Midu County; *XY* Xiangyun County; *EY* Eryuan County; *DL* Dali City; *WS* Weishan County; *JC* Jianchuan County; *CX* Chuxiong City; *NJ* Nanjian County; *GC* Gucheng County of Lijiang City; *YS* Yongsheng County

The selected villages were Lianyi (HQ1) and Jinsuo (HQ2) in Heqing County, Caizhuang (MD1) in Midu County, Xiaoqiao (XY1) in Xiangyun County, Qiandian (EY1) and Yongle (EY2) in Eryuan County, Liuguanchang (DL1), Wuxing (DL2), Machang (DL3), and Fengming (DL4) in Dali City, Dianzhong (WS1) in Weishan County, Huilong (JC1) in Jianchuan County, Cangling (CX1) and Luhe (CX2) in Chuxiong County, Biyuan (NL1) in Ninglang County, Dongyuan (GC1) in Lijiang City, and Yangwu (YS1) in Yongsheng County. Approximately 100 snails were collected from each sampling site. The habitat characteristics of *O. h. robertsoni* in the sampling site for each environmental type are presented in Additional file 1: Fig. S1.

### Extraction of total DNA from *O. hupensis*

The collected *O. h. robertsoni* snails were fed for 1 week, and we identified whether snails were infected with *S. japonicum* by observation of cercaria emergence from snails [27]. Thirty randomly selected *S. japonicum*-negative snails from each sampling site were used in this study. Genomic DNA was extracted from the head-foot muscle of each snail using the Qiagen extraction kit [Paisennuo Biotechnology (Shanghai) Co., Ltd.].

### Gene scan

Six microsatellite DNA polymorphic loci were selected from a previously published microsatellite loci library (i.e. P82, P84, T4-22, T5-11, T5-13, T6-27) [27]. Primer sequences and related information are presented in Table 2.

**Table 2:** Primers of the six microsatellite loci in *Oncomelania robertsoni*

Locus	Primer sequence (5ʹ → 3ʹ)	Repeat motif	Fluorescent labeling
P82 Pf	AAGAACTGCTCATACTGGAAAG	(GGA)_4_(GAA)_12_	FAM
P82 Pr	GTGGTGCCCCTACGACCT		
P84 Pf	GTTGCAGATTCCGAAAGA	(TACAT)_3_	HEX
P84 Pr	GATCAGGGGTTGTCCAGT		
T4-22 Pf	TATCCAAGAAGCCGAAAC	(CA)_10_	TAMRA
T4-22 Pr	GAGGAAAGCGAGGTAAGA		
T5-11 Pf	ACGCCAGTCTTGGTGTCA	(GT)_14_	FAM
T5-11 Pr	TACTTGGGCAGAAGGGTT		
T5-13 Pf	TAGTGGGACTTATTTGCTG	(GT)_14_…(GT)_6_	HEX
T5-13 Pr	AAGGCTGAGTGGTAGTTA		
T6-27 Pf	AATGACACCCCGAACAAA	(G)_13_(GT)_6_…(GT)_2_	TAMRA
T6-27 Pr	CACTTCTCAACTCCAACCT		

PCR amplification was performed. The amplification reaction system was 20 μl, including 1 μl template DNA, 1 μl forward fluorescent primer, 1 μl reverse primer, 2 μl 10*buffer, 0.5 μl dNTP, 0.5 μl Taq enzyme, and 14 μl DdH_2_0. The reaction conditions for PCR amplification were as follows: 95 °C, 5 min; 95 °C, 30 s, 62–52 °C, 30 s, 72 °C, 30 s, 10 cycles; 95 °C, 30 s, 52 °C, 30 s, 72 °C, 30 s, 25 cycles; 72 °C, 7 min for final extension. After the completion of PCR, a portion of the samples was subjected to agarose gel electrophoresis to determine the concentration and fragment size range of the samples. The samples were adjusted based on electrophoresis results, and 70% ethanol was added to a final volume of 50 μl. After thorough mixing, centrifugation was performed at 3700 rpm and 4 °C for 30 min, followed by sample purification. Ethanol was then removed after inversion. Finally, PCR products were mixed with LIZ500 and Hi-Di, denatured at 95 °C for 4 min and detected using an automated genetic analyzer (3730XL, ABI, USA).

### Analysis of microsatellite diversity

The length of the amplified fragments of microsatellite DNA loci were determined and subsequently exported as an Excel table for genetic diversity analysis.

First, genetic differentiation analysis was conducted within populations, calculating the number of alleles (Na), expected heterozygosity (He), observed heterozygosity (Ho), and fixation index (F) for each population at six microsatellite DNA loci, respectively. Hardy-Weinberg equilibrium (HEW) tests were also performed. The values of Na, Ho, and F represent the degree of population genetic differentiation, with higher values of Na and Ho and negative values of F indicating greater genetic diversity [28–30]. When He exceeds Ho, it suggests a lack of heterozygosity within the population [31, 32]. Additionally, the polymorphic information content (PIC) was calculated, with higher PIC values indicating greater genetic variation. Higher values represent higher polymorphism where the locus was classified based on their value (i.e. low polymorphic = < 0.25, moderately polymorphic = 0.25–0.50, and highly polymorphic = > 0.50) [33].

Second, genetic differentiation among populations was assessed using pairwise Fst, which measures the probability that two gametes randomly selected from two populations are homologous. The value ranged from 0 to 1, with a higher value representing greater genetic differentiation between the populations [25, 34].

Third, based on Nei's genetic distance, phylogenetic trees were constructed using the minimum evolution (ME) method, neighbor-joining (NJ) method, and unweighted pair-group (UPG) method. Based on genetic distance, principal coordinate analysis (PCoA) was performed to determine the genetic variation structure, and analysis of molecular variance (AMOVA) was conducted to analyze the sources of variation in the snail population.

All analyses were conducted using GenAlEx (version 6.5), MEGA (version 11), and Cervus (version 3.0.7) software. HEW test and AMOVA considered *P* value < 0.05 as statistically significant.

## Results

### Gene scan

A total of 507 specimens from the 17 sampled populations of *O. h. robertsoni* were scanned at the genetic level across six polymorphic loci of microsatellite DNA, resulting in 6084 microsatellite DNA loci data (Additional file 2: Dataset S2).

### Genetic differentiation within populations

As shown in Table 3, the Na of the 17 *O. h. robertsoni* populations ranged from 0 to 19, with a mean of 5. The CX2 and WS1 populations had the minimum and maximum average Na values, respectively. The mean ranges of He and Ho within populations were 0.506 to 0.761 and 0.443 to 0.792, respectively. The HQ2 and WS1 populations had the minimum and maximum mean Ho values, respectively. Most populations exhibited lower Ho than He, indicating a loss of heterozygosity. The mean of F within the populations ranged from – 0.801 to 0.211. The mean of PIC within population ranged from 0.411 to 0.757, with a mean of 0.557. Significant deviation from HWE was observed in 68 out of 102 (24.48%) possible single exact locus tests (*P* < 0.05). Except for the YS1 population, which had only one locus deviating from HWE, the remaining 16 populations had three to five loci deviating from HWE.

**Table 3:** Coefficients of genetic diversity of *Oncomelania hupensis robertsoni* at different loci

Population	Index	Microsatellite loci						Mean
		P82	P84	T4-22	T5-11	T5-13	T6-27	
Lianyi (HQ1)	Na	5	2	4	8	8	5	5.333
	Ho	0.267	0.033	0.433	0.967	1.000	0.967	0.611
	He	0.688	0.500	0.660^*^	0.838^*^	0.837^*^	0.760^*^	0.714
	F	0.612	0.933	0.343	− 0.153	− 0.195	– 0.271	0.212
	PIC	0.636	0.375	0.597	0.817	0.816	0.719	0.660
Jinsuo (HQ2)	Na	4	0	4	6	4	5	3.833
	Ho	0.100	0.000	0.100	0.967	0.900	0.967	0.506
	He	0.722	0.000	0.722	0.656^*^	0.598^*^	0.596^*^	0.549
	F	0.862	0.000	0.862	− 0.470	− 0.500	− 0.616	0.028
	PIC	0.671	0.000	0.671	0.599	0.518	0.516	0.496
Caizhuang (MD1)	Na	3	3	6	6	3	9	5.000
	Ho	0.133	0.067	0.233	1.000	0.967	1.000	0.567
	He	0.625	0.625	0.781	0.651^*^	0.517^*^	0.743^*^	0.657
	F	0.787	0.893	0.687	− 0.478	− 0.760	− 0.314	0.136
	PIC	0.555	0.555	0.748	0.594	0.400	0.706	0.593
Xiaoqiao (XY1)	Na	2	2	4	8	11	9	6.000
	Ho	0.000	0.000	0.667	1.000	1.000	0.933	0.600
	He	0.500	0.500	0.143^*^	0.621^*^	0.768^*^	0.629^*^	0.527
	F	NA	NA	− 1.899	− 0.576	− 0.308	− 0.421	− 0.801
	PIC	0.375	0.375	0.139	0.548	0.735	0.559	0.455
Qiandian (EY1)	Na	0	2	3	3	6	6	3.333
	Ho	0.000	0.100	0.133	1.000	1.000	1.000	0.539
	He	0.000	0.500	0.611	0.516^*^	0.707^*^	0.578^*^	0.485
	F	0.000	0.800	0.749	− 0.938	− 0.386	− 0.731	− 0.101
	PIC	0.000	0.375	0.535	0.399	0.667	0.488	0.411
Yongle (EY2)	Na	2	2	6	7	8	5	5.000
	Ho	0.333	0.033	0.900	1.000	1.000	1.000	0.711
	He	0.500^*^	0.500	0.767^*^	0.757^*^	0.818^*^	0.664^*^	0.668
	F	0.333	0.933	− 0.170	− 0.306	− 0.208	− 0.499	0.014
	PIC	0.375	0.375	0.727	0.721	0.795	0.599	0.599
Liuguanchang (DL1)	Na	2	2	6	7	6	9	5.333
	Ho	0.300	0.033	0.500	1.000	0.967	1.000	0.633
	He	0.500^*^	0.500	0.784^*^	0.771^*^	0.646^*^	0.806^*^	0.668
	F	0.400	0.933	0.384	-0.303	-0.440	-0.237	0.123
	PIC	0.375	0.375	0.753	0.741	0.589	0.779	0.602
Wuxing (DL2)	Na	2	0	5	5	7	2	3.500
	Ho	0.367	0.000	0.267	1.000	1.000	1.000	0.606
	He	0.500^*^	0.000	0.673	0.705^*^	0.729^*^	0.500^*^	0.518
	F	0.267	0.000	0.641	− 0.380	− 0.345	− 0.879	− 0.139
	PIC	0.375	0.000	0.617	0.660	0.687	0.375	0.452
Populations	Index	Microsatellite loci						Mean
		P82	P84	T4-22	T5-11	T5-13	T6-27	
Machang (DL3)	Na	2	0	7	6	5	2	3.667
	Ho	0.172	0.000	0.690	1.000	0.931	1.000	0.632
	He	0.500^*^	0.000	0.775^*^	0.773^*^	0.731^*^	0.500^*^	0.547
	F	0.655	0.000	0.110	− 0.288	− 0.293	− 1.000	− 0.163
	PIC	0.375	0.000	0.740	0.739	0.686	0.375	0.486
Fengming (DL4)	Na	0	2	6	6	10	6	5.000
	Ho	0.000	0.621	0.586	1.000	1.000	0.966	0.701
	He	0.000	0.500^*^	0.637^*^	0.777	0.822^*^	0.716^*^	0.575
	F	0.000	− 0.241	0.089	− 0.290	− 0.203	− 0.327	− 0.007
	PIC	0.000	0.375	0.573	0.741	0.800	0.672	0.527
Dianzhong (WS1)	Na	8	2	13	9	19	12	10.500
	Ho	0.633	0.167	0.933	0.933	0.933	0.967	0.761
	He	0.846^*^	0.500^*^	0.874^*^	0.783^*^	0.919^*^	0.830	0.792
	F	0.252	0.667	− 0.064	− 0.211	− 0.021	− 0.175	0.075
	PIC	0.828	0.375	0.862	0.756	0.913	0.809	0.757
Huilong (JC1)	Na	6	2	7	5	6	5	5.167
	Ho	0.433	0.033	1.000	0.967	0.933	1.000	0.728
	He	0.731^*^	0.500	0.670^*^	0.701^*^	0.690^*^	0.605^*^	0.650
	F	0.407	0.933	− 0.520	− 0.335	− 0.304	− 0.583	− 0.067
	PIC	0.687	0.375	0.612	0.650	0.636	0.525	0.581
Cangling (CX1)	Na	2	0	6	6	5	6	4.167
	Ho	0.300	0.000	0.967	1.000	1.000	1.000	0.711
	He	0.500^*^	0.000	0.681^*^	0.671^*^	0.548^*^	0.563^*^	0.494
	F	0.400	0.000	− 0.419	− 0.500	− 0.775	− 0.826	− 0.424
	PIC	0.375	0.000	0.621	0.610	0.445	0.468	0.420
Luhe (CX2)	Na	0	2	2	2	3	3	2.000
	Ho	0.000	0.067	1.000	1.000	1.000	1.000	0.678
	He	0.000	0.500	0.500^*^	0.500^*^	0.625^*^	0.531^*^	0.443
	F	0.000	0.867	− 0.879	− 0.879	− 0.560	− 0.779	− 0.446
	PIC	0.000	0.875	0.875	0.875	0.805	0.859	0.715
Biyuan (NL1)	Na	4	0	6	12	8	8	6.333
	Ho	0.167	0.000	0.900	1.000	1.000	1.000	0.678
	He	0.722	0.000	0.697^*^	0.810^*^	0.686^*^	0.799^*^	0.619
	F	0.762	0.000	− 0.254	− 0.229	− 0.413	− 0.233	− 0.073
	PIC	0.671	0.000	0.643	0.786	0.639	0.773	0.585
Dongyuan (GC1)	Na	0	4	8	6	6	5	4.833
	Ho	0.000	0.133	0.967	1.000	1.000	0.933	0.678
	He	0.000	0.656	0.732^*^	0.662^*^	0.657^*^	0.706^*^	0.569
	F	0.000	0.797	− 0.302	− 0.455	− 0.472	− 0.321	0.030
	PIC	0.000	0.605	0.691	0.602	0.599	0.654	0.525
Populations	Index	Microsatellite loci						Mean
		P82	P84	T4-22	T5-11	T5-13	T6-27	
Yangwu (YS1)	Na	2	0	7	10	16	11	7.667
	Ho	0.034	0.000	0.552	1.000	1.000	0.897	0.581
	He	0.500	0.000	0.736	0.869	0.879^*^	0.857	0.640
	F	0.931	0.000	0.281	− 0.151	− 0.142	− 0.049	0.174
	PIC	0.375	0.000	0.705	0.856	0.868	0.842	0.608
Mean	Na	2.588	1.471	5.882	6.588	7.706	6.353	5.098
	Ho	0.197	0.076	0.637	0.990	0.978	0.978	0.643
	He	0.461	0.340	0.673	0.709	0.716	0.670	0.595
	F	0.631	0.752	− 0.021	− 0.408	− 0.372	− 0.486	− 0.074
	PIC	0.393	0.296	0.653	0.688	0.682	0.630	0.557

*HQ* Heqing County; *MD* Midu County; *XY* Xiangyun County; *EY* Eryuan County; *DL* Dali City; *WS* Weishan County; *JC* Jianchuan County; *CX* Chuxiong City; NJ, Nanjian County; *GC* Gucheng County of Lijiang City; *YS* Yongsheng County; *Na* number of alleles; *Ho* observed heterozygosity; *He* expected heterozygosity; *F* fixation index; *PIC* polymorphic information content

^*^Significant deviation from Hardy-Weinberg equilibrium

In summary, there is a high degree of genetic differentiation in snail samples of *O. h. robertsoni*. Among these, the highest genetic differentiation was observed in the WS1 population.

### Genetic differentiation among populations

As shown in Table 4, across the 17 *O. h. robertsoni* populations, the lowest estimates of pairwise Fst were observed between WS1 population and JC1 population, with 0.051, whereas the highest estimates of pairwise Fst were observed between CX2 population and DL2 population, with 0.379.

**Table 4:** Pairwise Fst among *Oncomelania hupensis robertsoni* populations

Population	HQ1	HQ2	MD1	XY1	EY1	EY2	DL1	DL2	DL3	DL4	WS1	JC1	CX1	CX2	NL1	GC1	YS1
HQ1	0.000																
HQ2	0.181	0.000															
MD1	0.111	0.244	0.000														
XY1	0.175	0.316	0.181	0.000													
EY1	0.218	0.375	0.224	0.171	0.000												
EY2	0.067	0.260	0.119	0.160	0.199	0.000											
DL1	0.079	0.244	0.103	0.142	0.172	0.057	0.000										
DL2	0.219	0.335	0.242	0.282	0.318	0.149	0.193	0.000									
DL3	0.191	0.313	0.223	0.250	0.278	0.170	0.157	0.214	0.000								
DL4	0.212	0.313	0.195	0.264	0.274	0.210	0.182	0.319	0.299	0.000							
WS1	0.080	0.207	0.091	0.144	0.193	0.083	0.082	0.187	0.169	0.130	0.000						
JC1	0.124	0.256	0.124	0.164	0.189	0.087	0.085	0.184	0.163	0.166	0.051	0.000					
CX1	0.168	0.327	0.229	0.330	0.366	0.187	0.197	0.276	0.240	0.324	0.194	0.230	0.000				
CX2	0.155	0.349	0.240	0.300	0.321	0.214	0.220	0.379	0.322	0.309	0.204	0.244	0.291	0.000			
NL1	0.201	0.237	0.217	0.301	0.343	0.233	0.217	0.302	0.294	0.281	0.178	0.238	0.313	0.362	0.000		
GC1	0.196	0.300	0.236	0.292	0.322	0.228	0.232	0.337	0.342	0.289	0.173	0.222	0.363	0.339	0.277	0.000	
YS1	0.169	0.227	0.196	0.267	0.337	0.222	0.218	0.292	0.273	0.310	0.186	0.221	0.284	0.335	0.242	0.294	0.000

*HQ* Heqing County; *MD* Midu County; *XY* Xiangyun County; *EY* Eryuan County; *DL* Dali City; *WS* Weishan County; *JC* Jianchuan County; *CX* Chuxiong City; *NJ* Nanjian County; *GC* Gucheng County of Lijiang City; *YS* Yongsheng County; *Na* number of alleles; *Ho* observed heterozygosity; *He* expected heterozygosity; *F* fixation index; *PIC* polymorphic information content

### Population structure

**Fig. 2:**
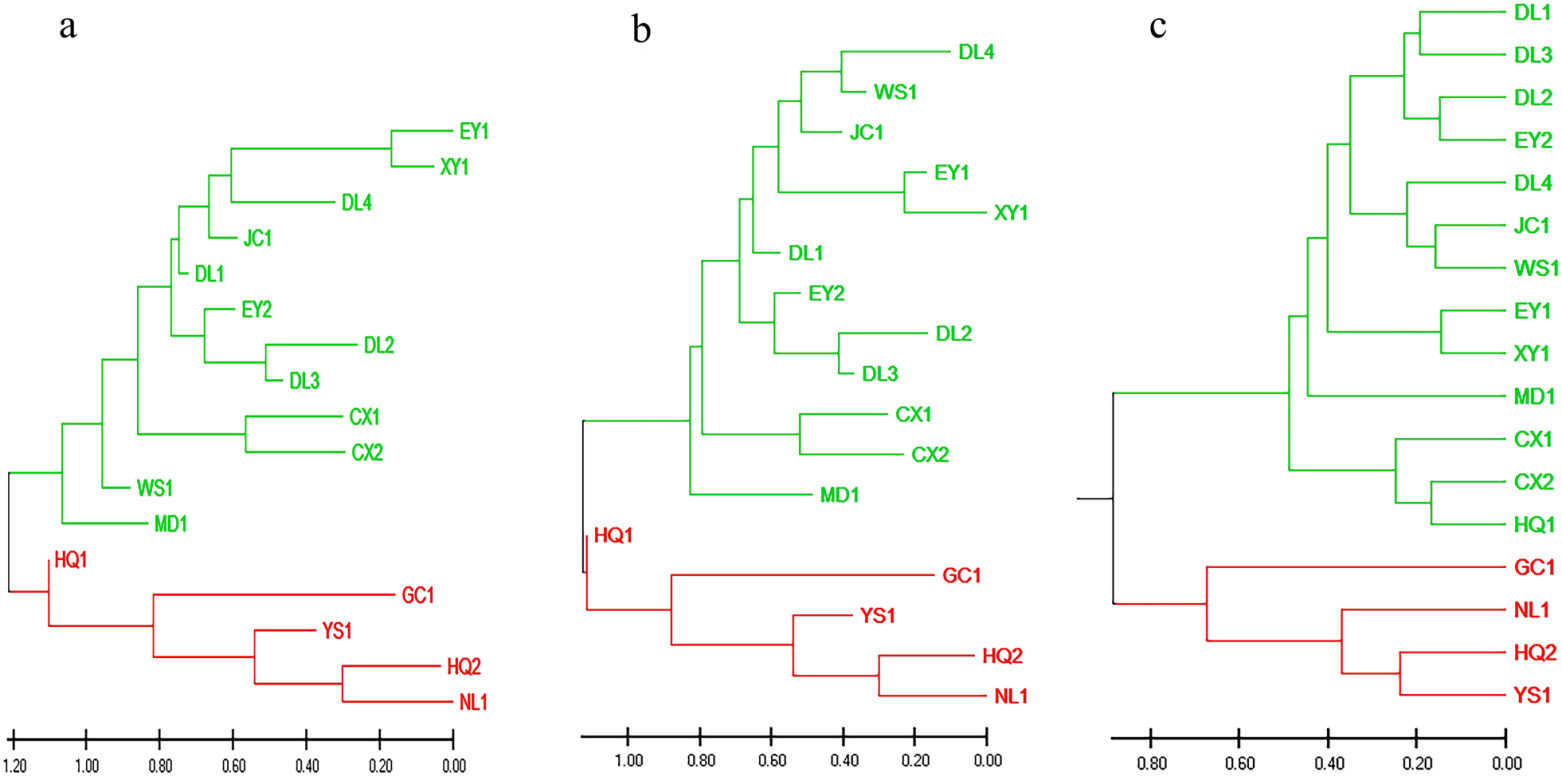
Phylogenetic tree constructed by minimum evolution (ME) method (**a**), neighbor-joining (NJ) method (**b**), and unweighted pair-group (UPG) method (**c**)

**Fig. 3:**
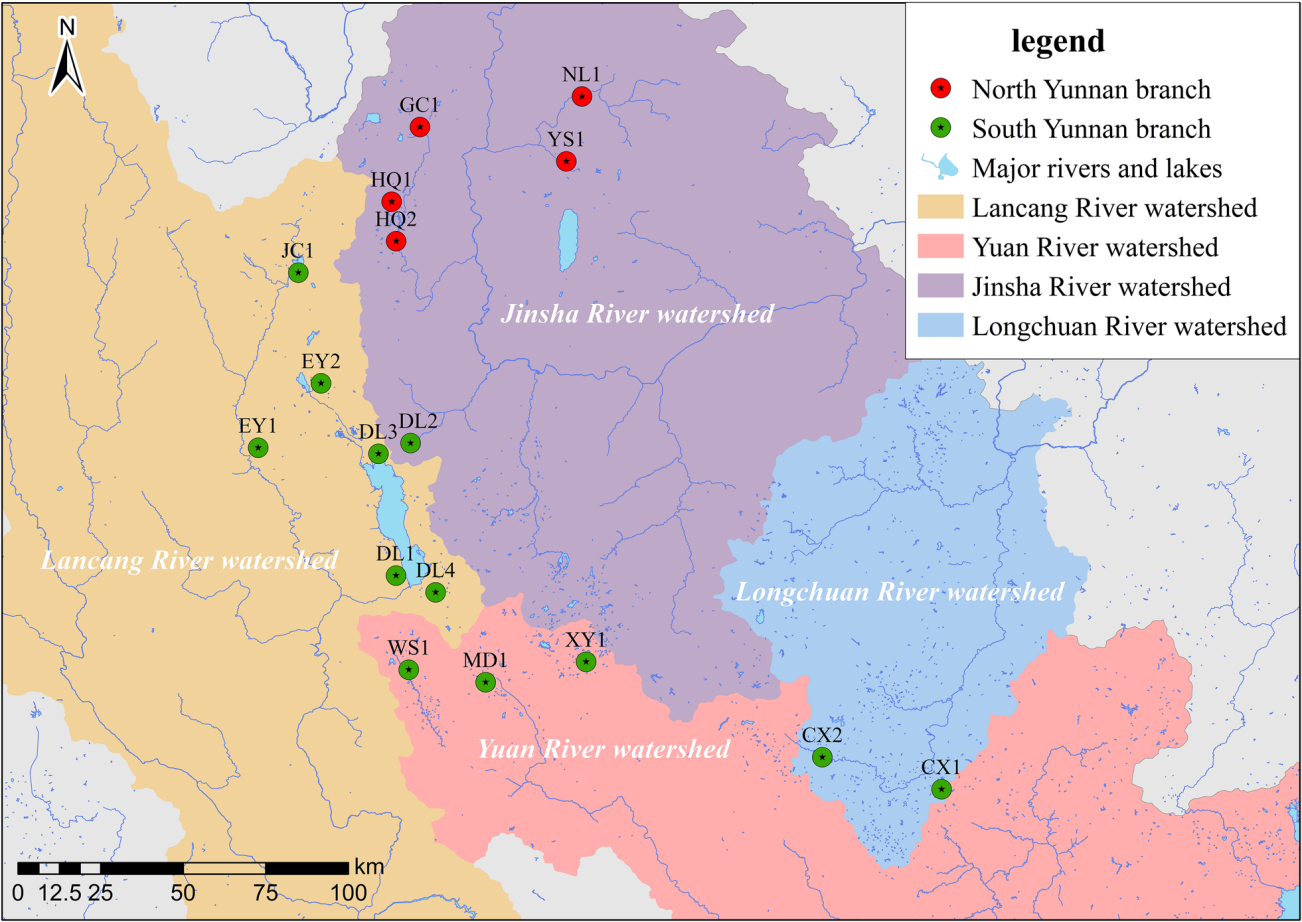
Geographical locations of north-south Yunnan branch of *Oncomelania hupensis robertsoni*

The phylogenetic trees constructed using the ME, NJ, and UPG methods (Fig. 2a–c) consistently divided the 17 *O. h. robertsoni* populations into two major branches, tentatively named “Yunnan North Branch” and “Yunnan South Branch,” based on their geographical locations. The topological structures of the trees constructed using ME and NJ methods were consistent, whereas those resulting from the UPG method differed in the clustering of the HQ1 population. Based on the ME and NJ cluster analyses, the Yunnan South Branch included populations distributed in the Lancang River Watershed (i.e. JC1, EY1, EY2, DL1, DL2, DL3, and DL4), Yuan River Watershed (i.e. WS1, MD1, and XY1), and Longchuan River Watershed (i.e. CX1 and CX2), located in central and southern Yunnan Province. The Yunnan North Branch included populations distributed in the Jinsha River Watershed (i.e. NJ1, YS1, GC1, HQ1, and HQ2), located in northern Yunnan Province. However, based on UPG method, the HQ1 population was clustered in the Yunnan South Branch by UPG method. There are clear geographic barriers between the Jinsha River Watershed and the Lancang River, Yuan River, and Longchuan River Watershed, as shown in Fig. 3.

**Fig. 4:**
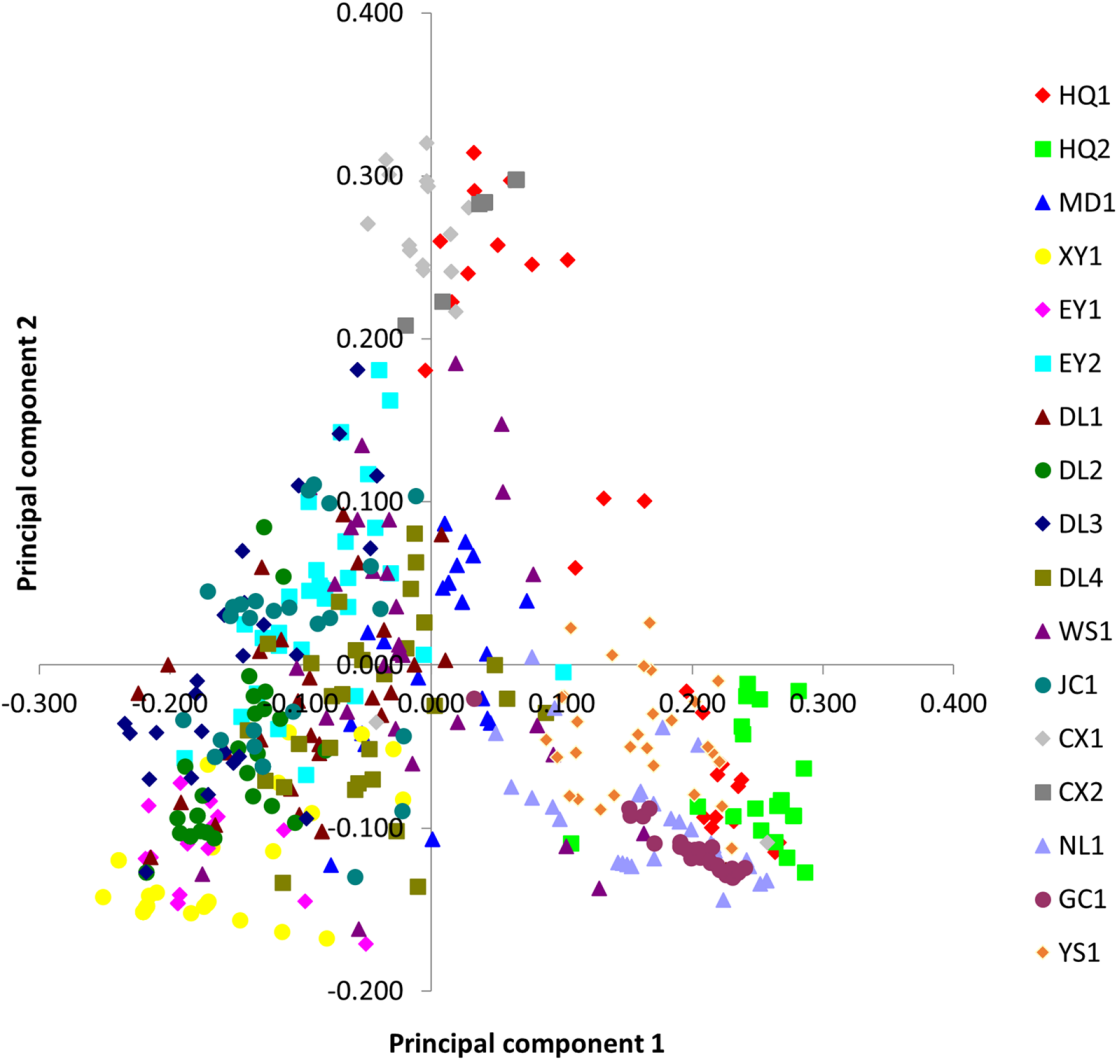
A two-dimensional plot of the principal coordinate analysis (PCoA) of microsatellite data showing the clustering of *Oncomelania hupensis robertsoni*

The PCoA (Fig. 4) based on the covariance of the genetic distance matrix revealed that individuals from each population were relatively concentrated, with some populations such as HQ1 and MD1 showing relatively dispersed individuals, corresponding approximately to the branches in the constructed phylogenetic tree.

AMOVA results (Table 5) indicated that the source of variation was mainly within *O. h. robertsoni* populations, accounting for 88% of the total variation, compared with variation among *O. h. robertsoni* populations, which accounted for 12%. Fst among the 17 *O. h. robertsoni* populations was 0.170 (*P* < 0.001), indicating significant genetic differentiation between at least two populations.

**Table 5:** Analysis of molecular variance (AMOVA) for *Oncomelania hupensis robertsoni* populations

Source of variation	Degree of freedom	Sum of squares	Mean square	Percentage of variation (%)	Fixation index
Among populations	16	401.418	25.089	12	Fst = 0.170^*^
Within populations	507	1510	2.978	88	

^*^P < 0.001

## Discussion

Eliminating schistosomiasis remains a challenging public health endeavor worldwide [35, 36]. Given the sustainable parasitism of *S. japonicum* in 42 nonhuman mammalian species, control strategies for this parasite infection in wild animals (e.g. rodents) might not be practical [37, 38]; thus, monitoring and controlling its only intermediate host snail to interrupt the transmission chain is a more feasible approach [39]. However, the natural environment of hilly areas of schistosomiasis endemism in China is complex, with scattered snail breeding grounds, making it difficult to further compress the snail’s breeding areas and rendering snail monitoring less effective [40]. In this study, we collected 17 representative *O. h. robertsoni* snail populations from hilly endemic schistosomiasis areas in Yunnan Province of China and analyzed the genetic diversity of this population using microsatellite DNA markers, which are considered suitable, neutral Mendelian markers [41]. Our results may provide theoretical support for the targeted monitoring and control of *O. h. robertsoni* in such regions.

In this research, the Ho of the 17 *O. h. robertsoni* populations ranged from 0.506 to 0.761 (all exceeding 0.500), with most populations exhibiting higher Ho than He and deviating from HWE at six microsatellite DNA polymorphic loci. These results indicated a significant deficiency of heterozygotes in the *O. h. robertsoni* population, reflecting a relatively high degree of genetic differentiation [42, 43]. High genetic differentiation could be associated with higher fitness of hosts and decreased susceptibility to parasite infection [44, 45], whereas the causes of low diversity may be related to low degrees of habitat disturbance, large population sizes, or persistently habituated sites [24]. We hypothesize that the observed high diversity of *O. h. robertsoni* may mainly result from one or multiple factors mentioned above. In fact, the *O. h. robertsoni* breeding environment in Yunnan Province is complex, presenting a patchy distribution, and snail control measures mainly rely on spraying of molluscides, such as niclosamide and metaldehyde [46]. The effectiveness of such an approach is limited by geographical constraints and higher water source requirements [47], resulting in relatively low habitat disturbance and a large population size.

Additionally, in this study, the average PIC in the 17 *O. h. robertsoni* population ranged from 0.411 to 0.757. In contrast, another study using the same microsatellite loci for *O. h. hupensis* population reported average PIC values ranging from 0.511 to 0.850 [27]. This suggests that the level of genetic diversity previously reported for the *O. h. hupensis* population may be higher than that for the study *O. h. robertsoni* population.

Among the 17 *O. h. robertsoni* populations analyzed, the WS1 population had the highest average Na, He, Ho, and PIC, indicating the highest level of genetic differentiation, possibly because of a larger population size [13]. In contrast, the CX2 population had the lowest mean number of alleles, the HQ2 population had the lowest Ho, and the EY1 population had the lowest PIC, indicating relatively lower genetic differentiation. Low host diversity can be associated with increased susceptibility to parasite infection [48, 49]. For example, in the Senegal River Basin, perturbations (i.e. construction of the Diama Dam) in population genetics that lead to decreased intrapopulation diversity could have contributed to the major *Schistosome mansoni* outbreak [50, 51]. These findings may provide important information for targeted formulation of schistosomiasis prevention and control measures.

The pairwise Fst values ranged from 0.051 (between WS1 population and JC1 population) to 0.379 (between CX2 population and DL2 population), indicating a certain degree of genetic differentiation among all pairwise comparisons of the *O. h. robertsoni* populations. Notably, compared with those from the marshland and/or lake areas endemic for schistosomiasis in China, the pairwise Fst values reported here were relatively elevated. This suggested a heightened genetic variation among snails in these hilly terrains. AMOVA revealed that most of the variation was within populations in *O. h. robertsoni*, i.e. close consanguinity within rather than among populations.

The phylogenetic tree results showed that the snails in this region could be divided into two branches: the northern branch in the Jinsha River Watershed and the southern branch in the Lancang River, Yuanjiang, and Longchuan River Watersheds. This distribution pattern correlates with river basins rather than presenting an island-like distribution. However, to the best of our knowledge, the difference of morphology or biology between the northern and southern branch has not been investigated so far; this merits further research attention. Notably, the HQ1 population, located at the junction of the Jinsha River and Lancang River Watersheds, shows clustering results using the ME and NJ methods that differ from those obtained using the UPG method, and this population showed relatively scattered positions in the PCoA plot. This may be due to the limited representativeness of the six microsatellite loci selected in this study, suggesting the need for further verification with increased sampling points and microsatellite loci.

Furthermore, the PIC value is directly proportional to both the abundance of heterozygotes and the amount of genetic information, with higher PIC values indicating a larger proportion of heterozygotes and richer genetic information [52, 53]. Among the six loci used in this study, all but two, P28 and P48, were highly polymorphic (all PIC > 0.5), with the mean of PIC for P28 and P48 being 0.393 and 0.296, respectively. However, previous research using the P28 and P48 loci for genetic differentiation of snails in marshland and/or lake endemic areas showed them to be high polymorphic. This suggested significant differences in P28 and P48 between lake- and hill-type snail populations, making them important markers for distinguishing between these types. However, they might not be suitable for studying subspecies of *O. hupensis* in hilly regions of China, providing valuable information for future genetic differentiation studies in *O. hupensis* snail populations.

Considering that only six microsatellite DNA polymorphic loci were analyzed in this study for determining genetic diversity, we therefore recommend the use of additional highly polymorphic microsatellite markers not only for *O. hupensis* but also for *Bulinus* species (*Bulinus* sp.), the sole intermediate host of *Schistosome mansoni* and *Biomphalaria* species (*Biomphalaria* sp.), the sole intermediate host of *Schistosome haematobium*, to be used in future studies for more precise analysis. This study, based on microsatellite markers, may provide genetic evidence to help monitoring and control of snails.

## Conclusions

This study, using microsatellite DNA markers, first demonstrated that the *O. h. robertsoni* snail populations generally had relatively high genetic diversity, and the variation mainly originates from within rather than among populations; the snails within populations have closer consanguinity. The notable north-south genetic differentiation of snail populations in this region was likely to be associated with specific watersheds. These findings provided crucial information for understanding the genetic structure and distribution patterns of *O. h. robertsoni* in hilly regions of China. Ultimately, our results might also have implications for the development of more effective strategies for the control of schistosomiasis in these regions.

Additionally, the microsatellite DNA loci P82 and P84 showed relatively low polymorphism in this study, suggesting that these loci were not be suitable for classification study of *O. hupensis* in hilly regions. This finding has significance for subsequent investigations into the genetic diversity of *O. hupensis* populations.

### Supplementary Information

**Additional file 1: Fig. S1.** Habitat characteristics of *Oncomelania hupensis robertsoni* in sampling site for each environmental type.

**Additional file 2: Dataset S2.** Microsatellite DNA loci data of the samples tested.

## Data Availability

All data other than the ones included in the main manuscript are available as Additional file 2: Dataset S2.

## Abbreviations

STR: Short nucleotide tandem
SSR: Imple sequence repeat
*O. h. robertsoni*: *Oncomelania hupensis robertsoni*
Na: Number of alleles
He: Expected heterozygosity
Ho: Observed heterozygosity
F: Fixation index
HEW: Hardy–Weinberg equilibrium
PIC: Polymorphic information content
ME: Minimum evolution
NJ: Neighbor-joining
UPG: Unweighted pair-group
PCoA: Principal coordinate analysis
AMOVA: Analysis of molecular variance
