# Supplementary material for: The genetic diversity of Oncomelania hupensis robertsoni, intermediate hosts of Schistosoma japonicum in hilly regions of China, using microsatellite markers
Source: Parasit Vectors. 2024 Mar 21;17:147. doi: 10.1186/s13071-024-06227-3 (PMC10956175; doi:10.1186/s13071-024-06227-3)
Supplement: Supplementary file 1 — Additional file 1: Fig. S1. Habitat characteristics of Oncomelania hupensis robertsoni in sampling site for each environmental type. [file 13071_2024_6227_MOESM1_ESM.pdf]

a

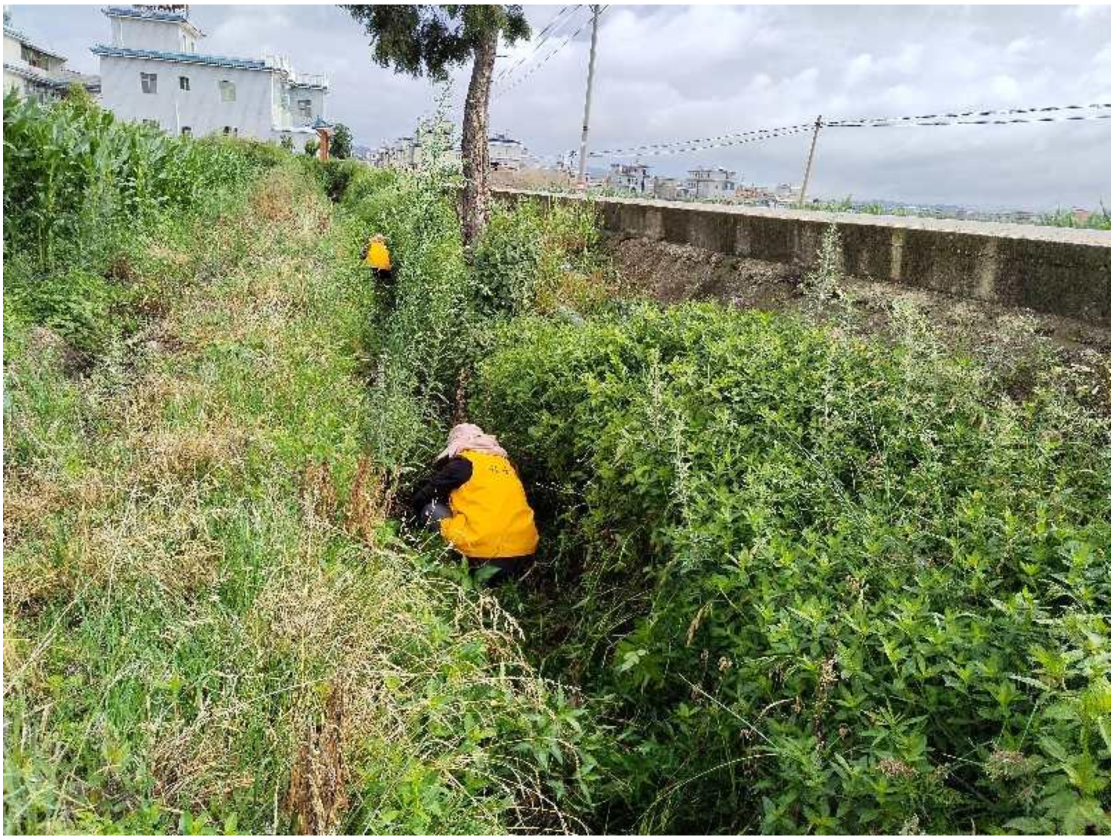

b

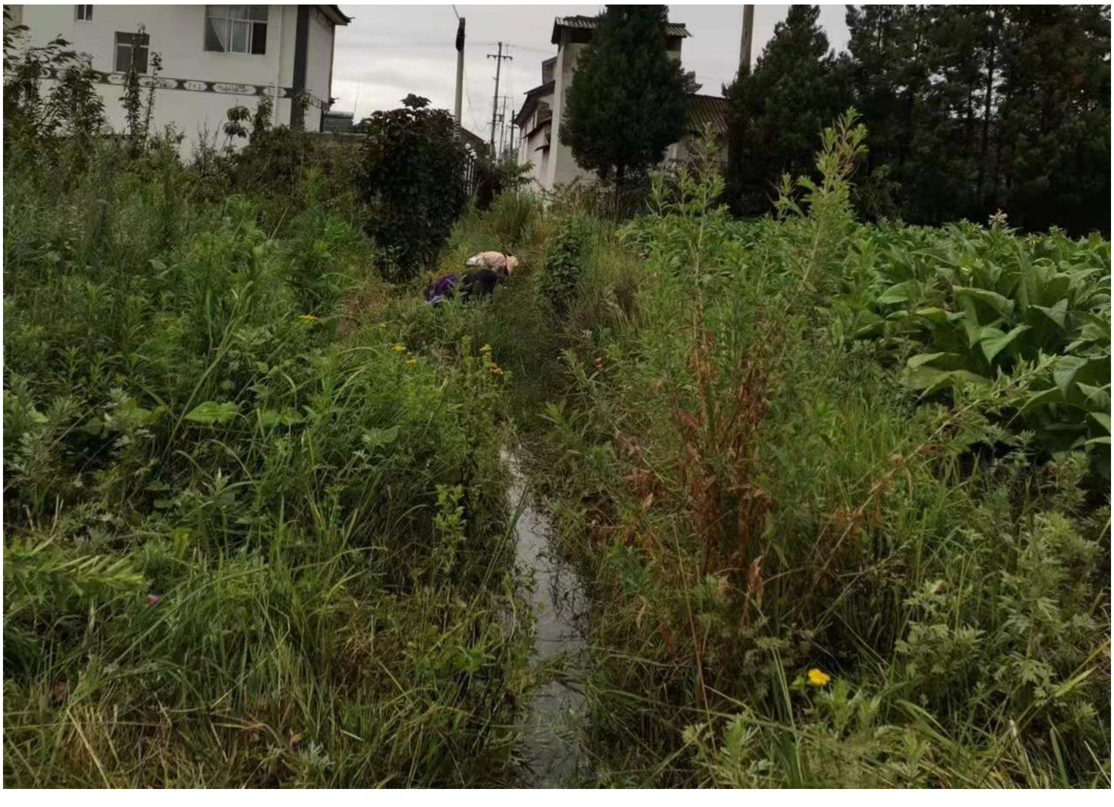

c

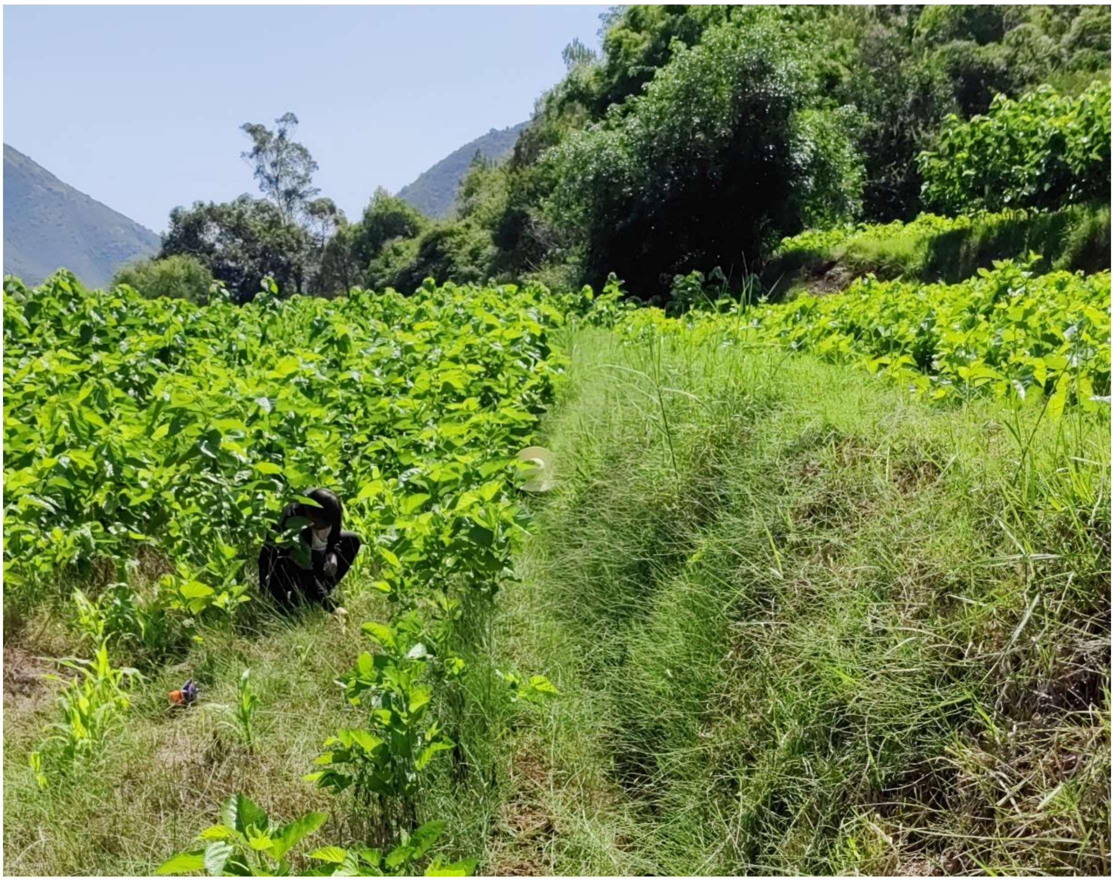

d

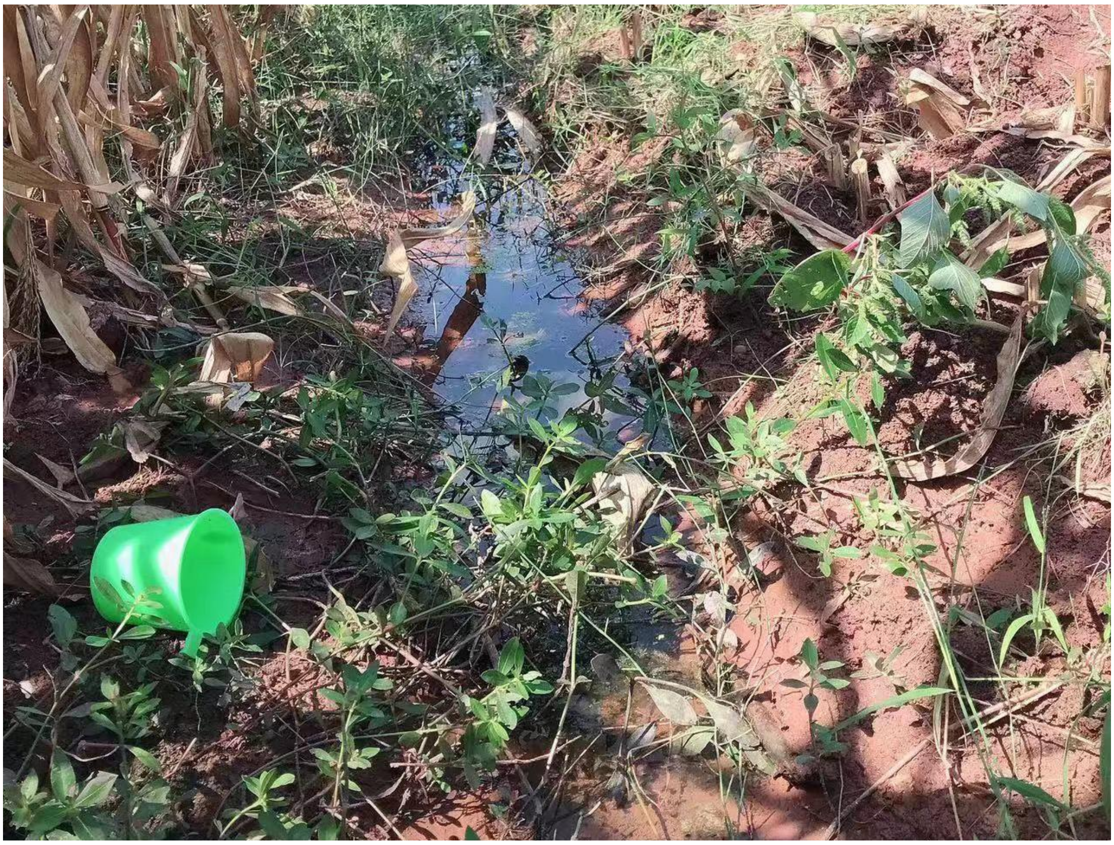

e

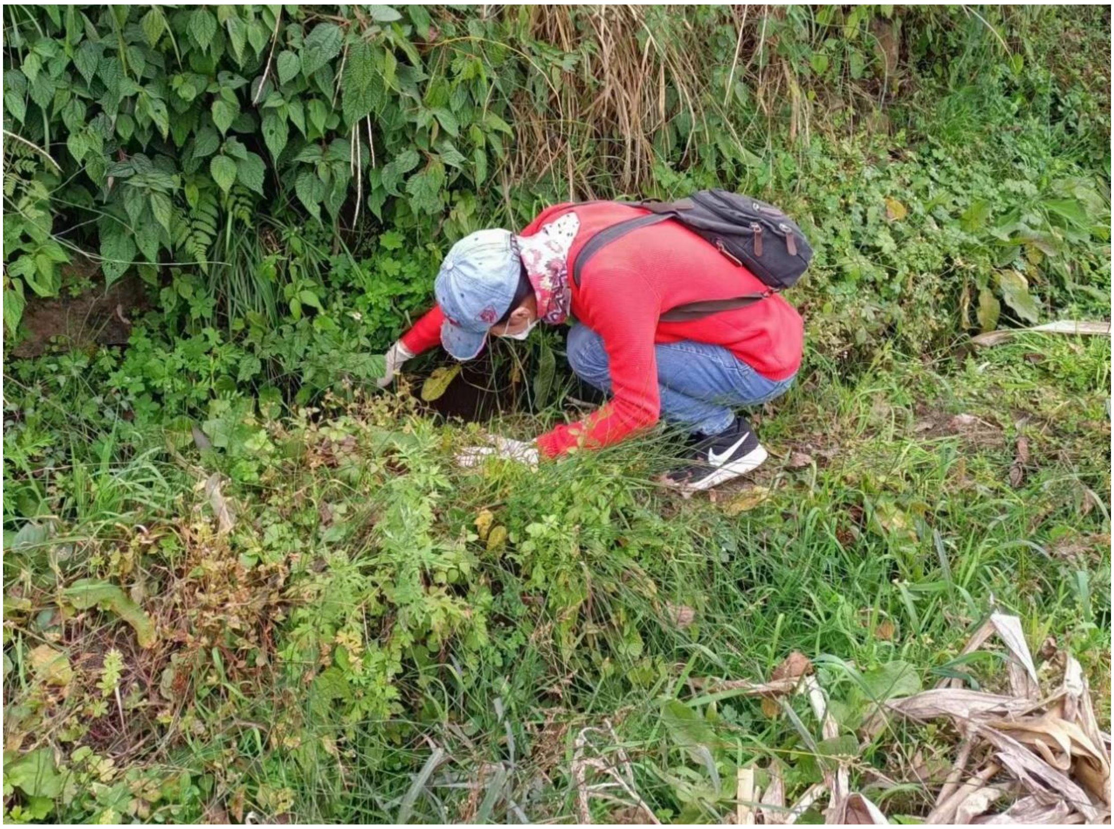

f

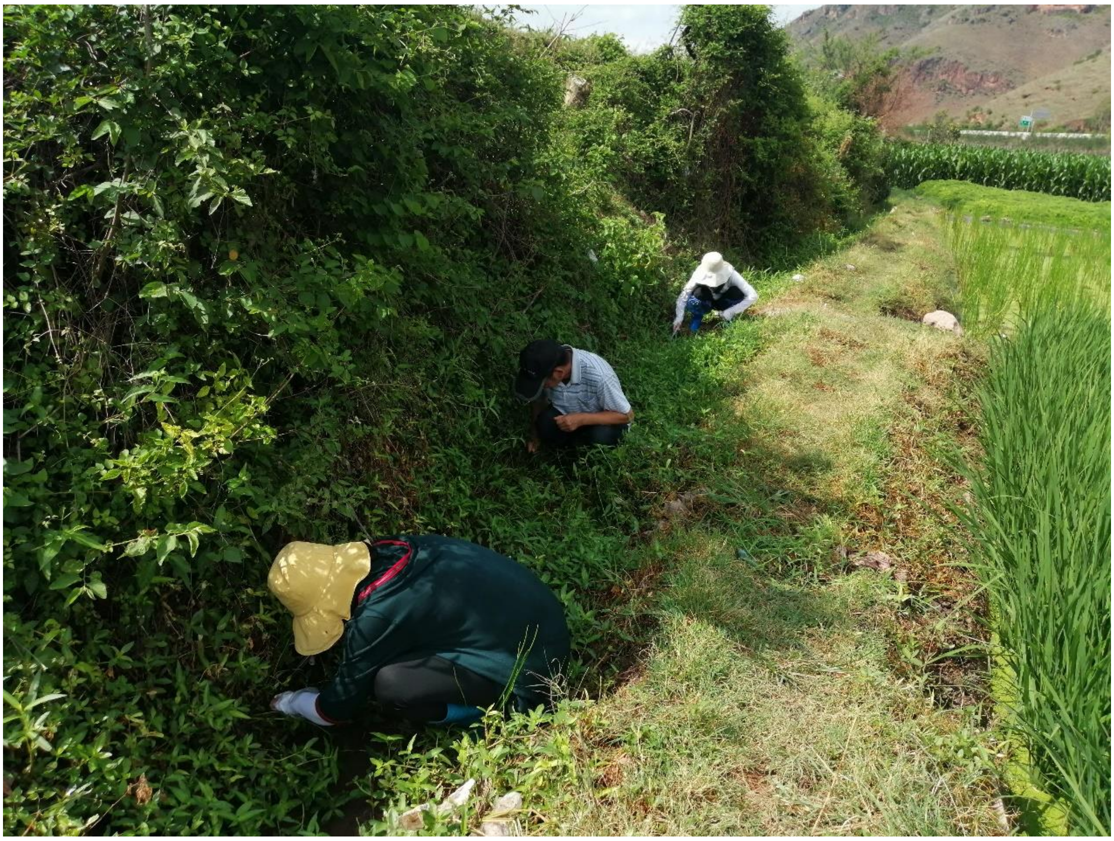

g

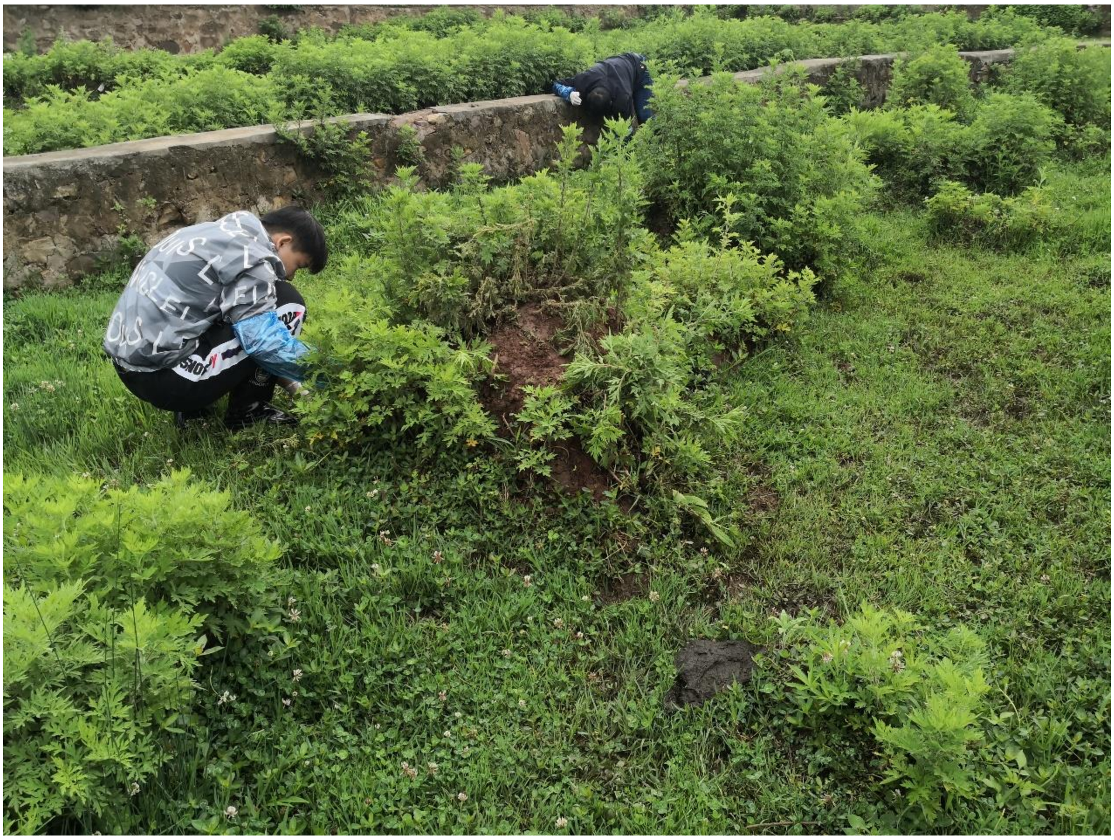

h

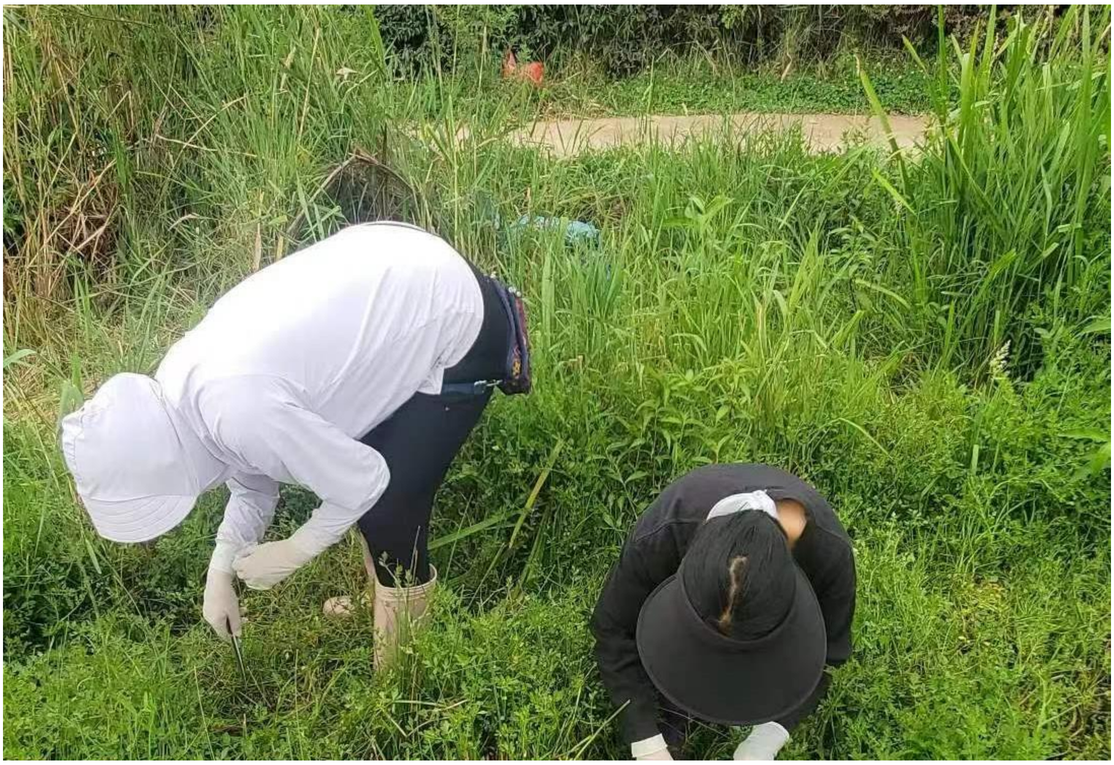

i

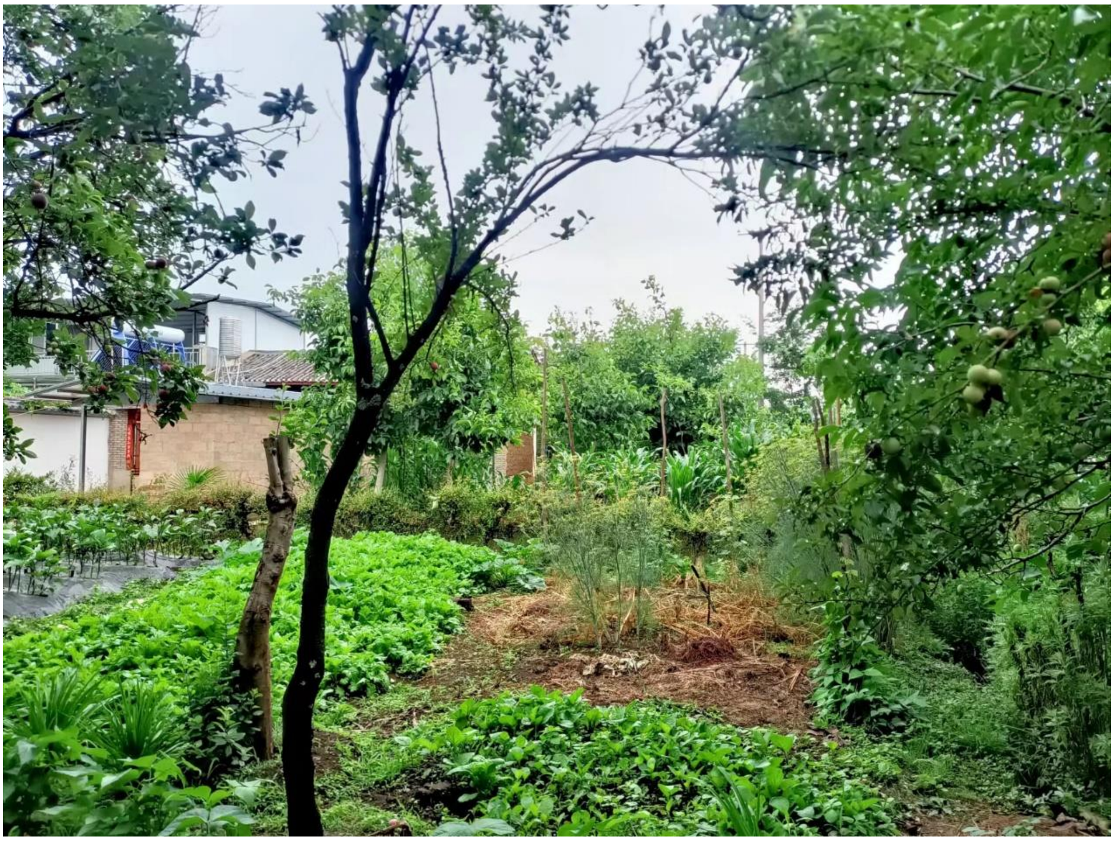

eFig.1 Habitat characteristics of *O. h. robertsoni* in sampling site for each environmental type. **a** Sampling site of XY1 (ditch). b sampling site of EY2 (ditch). **c** Sampling site of DL2 (ditch). **d** Sampling site of WS1 (ditch). **e** Sampling site of NL1 (ditch). **f** Sampling site of YS1 (ditch). **g** Sampling site of CX1 (grassland). **h** Sampling site of GC1 (grassland). **i** Sampling site of JC1 (vegetable field).
